# Human lung organoids model for assessing host response to *Mycobacterium tuberculosis* infection

**DOI:** 10.3389/fcimb.2026.1725344

**Published:** 2026-02-10

**Authors:** Chaofan Li, Pengfei Zhong, Zhimin Yun, Leiming Fang, Meida Xiang, Qisheng Su, Jiru Wang, Hebing Chen, Zhi Chen, Liang Yue, Yingxia Tan

**Affiliations:** 1 Academy of Military Medical Sciences, Beijing, China; 2 Department of Tuberculosis Medicine, Eighth Medical Center, PLA General Hospital, Beijing, China; 3 Department of Clinical Laboratory, The First Affiliated Hospital of Guangxi Medical University, Nanning, China; 4 Faculty of Medicine, Dalian University of Technology, Dalian, China

**Keywords:** host-pathogen interactions, innate immune response, lung organoids, TLR4-NF-κB pathway, tuberculosis

## Abstract

**Introduction:**

Airway and alveolar epithelial cells serve as the primary defense in the lower respiratory tract, yet their exact role in *Mycobacterium tuberculosis (Mtb)* infection is incompletely understood. Given that *Mtb* is a human-restricted pathogen, a representative human model is required. Lung organoids (LOs), which are composed of various epithelial cells, mesenchymal cells, and extracellular matrix, facilitate the investigation of bacterial infections.

**Methods:**

In this study, we established an *Mtb* infection model using human induced pluripotent stem cells (iPSCs)-derived LOs.

**Results:**

Prolonged infection led to the gradual invasion of *Mtb* from the periphery to the interior of the organoid, leading to decreased viability and the induction of fibrotic responses. Transcriptomic and protein analyses suggest that Mtb infection triggered a TLR4/NF-κB-associated inflammatory response. Additionally, the elevation of antimicrobial peptides and the release of diverse pro-inflammatory cytokines and chemokines were noted in the infected LOs.

**Conclusion:**

These findings emphasize the potential role of LOs in host defense and demonstrate that the *Mtb*-infected lung organoid model provides a novel platform for elucidating the role of pulmonary structural cells in tuberculosis pathogenesis. Furthermore, this model opens new avenues for the development of molecular therapeutic strategies.

## Introduction

Tuberculosis (TB) is a chronic infectious disease caused by *Mtb*, an obligate human pathogen, resulting in millions of deaths annually (Bagcchi, 2023). Human lungs are the primary target organ of *Mtb*, with pulmonary TB being the predominant clinical manifestation (Scriba et al., 2024). *Mtb* primarily enters the lower respiratory tract via aerosols, where it interacts with host cells in the bronchioles and alveoli. The proximal and distal airway epithelia, along with alveolar macrophages, serve as the initial barrier against the bacteria. Upon breaching this mucosal barrier, the initial host-pathogen interactions are critical for disease outcome. However, the establishment of infection has been traditionally attributed primarily to the phagocytosis of *Mtb* by alveolar macrophages (Scordo et al., 2016; Ryndak and Laal, 2019; Ahmad et al., 2022). The role of pulmonary structural cells, particularly the respiratory epithelium, in the initial stages of *Mtb* infection remains incompletely understood. Increasing evidence suggests that airway epithelia and alveolar cells, along with their innate immune defense mechanisms, play crucial roles in controlling TB (McDonough and Kress, 1995). It is noteworthy that lung epithelial cells may create a permissive niche for *Mtb* replication, potentially facilitating its survival. Compared to macrophages, alveolar epithelial cells exhibit lower stress levels in response to *Mtb* phagocytosis (Ryndak et al., 2015). Even though their role is so important, a major obstacle to elucidating the precise function of the human respiratory epithelium in TB pathogenesis is the lack of a physiologically relevant *in vitro* human model system. Common models, such as two-dimensional cell cultures or animal models, lack the complexity of interactions seen in humans, failing to accurately represent tuberculosis infection and dissemination. Therefore, a model that mimics the human organism is necessary. Organoids have shown promise in replicating human disease. Airway organoids from adult stem cells serve as a model for studying early mycobacterial infection mechanisms, revealing dynamic transcriptional changes in response to *Mtb* challenge, including cytokine, antimicrobial peptide, and mucin gene network modulation (Iakobachvili et al., 2022). However, this model lack of alveolar structures that *Mtb* most frequently encounters, means they fail to fully recapitulate the pulmonary epithelium’s response to the pathogen. In contrast, lung organoids (LOs) derived from human induced pluripotent stem cells (iPSCs) have the capacity to self-organize and include alveolar epithelial type II and I cell. Thus, they can mimic the niche where *Mtb* infection predominantly occurs, making them suitable for studying infectious diseases (Miller et al., 2019; Porotto et al., 2019).

In this study, we developed an *Mtb* infection model by exposing a human iPSCs-derived LOs to the virulent H37Rv strain to explore early host-pathogen interactions. We observed that *Mtb* initially adhered to the organoid periphery and was progressively internalized, marking a key step in the infection process. Our findings indicate that this interaction may triggers an innate immune response characterized by the upregulation of Toll-like receptor 4 (TLR4) and activation of the NF-κB signaling pathway, resulting in the secretion of pro-inflammatory cytokines and other immune mediators. Additionally, the infected LOs displayed pathological features, including the onset of fibrosis and a reduction in cell viability. Collectively, this human organoid-based TB model recapitulates critical early events of *Mtb* infection *in vitro*, providing a valuable platform to elucidate molecular mechanisms and designing early intervention strategies.

## Materials and methods

### Bacterial strains and cell culture

*Mtb* H37Rv was cultured on Middle Brook 7H11 agar basal medium (LA7240, Solaibio) supplemented with 10% OADC and 0.5% glycerol in a constant temperature incubator at 37 °C. When the bacterial growth reached the vigorous stage, the bacteria were transferred to a turbidity tube containing 2 mL PBS (Servicebio, G0002) using a bacterial loop, and the bacterial clumps were dispersed and quantified using an ultrasonic counter, and then the cell suspension was adjusted to 1 MCF with PBS. Subsequently, an appropriate volume of the bacterial suspension was inoculated into 24-well plates containing 1,000 µL of antibiotic-free medium to achieve a final bacterial concentration of 1×10^7^ CFU/mL per well.

### Human lung organoid generation

Human LOs culture was performed according to the previously described protocol (Leibel et al., 2019; Leibel et al., 2021). Briefly, human iPSCs were digested into single cells using Accutase (Gibco, A1110501), 100 ng/mL activin A (R&D Systems, P08476), 2 μM CHIR99021 (Selleckchem, S1263), and 10 μM Y27632 (Selleckchem, S6390) were used to induce differentiation to the DE stage for 5 days, and then induced to the AFE stage for 3 days in DMEM medium (Gibco, C11965500BT) containing 50 μg/mL L-ascorbic acid (Sigma-Aldrich, A4544) and 0.4 mM monothioglycerol (Sigma-Aldrich, M6145) supplemented with 10 μM SB431542 (R&D Systems, 1614) and 2 μM Dorsomorphin (Selleckchem, S7840). To form LPCs, the small molecule compounds were replaced with 10 ng/mL BMP4 (R&D Systems, 314-BP-050/CF), 50 nM Retinoic acid (RA, Sigma-Aldrich, R2625) and 3 μM CHIR99021 and incubated for a further 10 days. LPC cells were dissociated by Accutase, mixed with 20,000 cells per 50 uL Matrigel (354230, Corning) gel, and seeded in a pre-warmed 24-well plate. IMDM (Gibco, 12440053) containing 50 ng/mL FGF7 (R&D Systems, 251-KG-050), 100 ng/mL FGF10 (R&D Systems, 345-FG-250), 3 μM CHIR99021 and 50 ng/mL EGF (R&D Systems, 236-EG-200) was added [supplemented with BSA (Sigma-Aldrich, V900933), 1× N2 (Gibco, 17502048), 1× B27 (Gibco, 12587010), monothioglycerol and L-ascorbic acid] to form small spherical organoids. After 5–6 days, 50 nM RA and 20ng/mL VEGF (R&D Systems, DVE00) were added to induce the 3D spheroids to form vacuolar LOs for 6 days, and then differentiated and matured for 6 days under the continuous induction of 50 nM dexamethasone (Sigma-Aldrich, 265005), 100 μM cAMP (Sigma-Aldrich, 20-198) and 100 μM IBMX (Sigma-Aldrich, I5879).

### *Mtb* infected LOs model

After the LOs matured, they were cultured for another 2 days in antibiotic-free medium. To establish infection, 4 °C DPBS (Gibco, 14190144) and a trimmed wide-mouth 1 mL pipette were used to repeatedly blow the Matrigel to separate the organoids from the Matrigel while minimizing damage to the organoids. The suspension containing the organoids was transferred to a 15 mL centrifuge tube and centrifuged at 4 °C, 800 rpm for 3 minutes. The supernatant and Matrigel were removed, resuspended in antibiotic-free medium. Organoids ranging in diameter of 300-500 µm were selected based on observation under bright-field microscopy. These selected organoids were randomly divided into the experimental groups prior to infection. The selected organoids were inoculated into ultra-low adhesion 24-well plates (8–10 LOs of uniform size were visible to the naked eye per well). 1×10^7^ CFU/mL *Mtb* suspension was added to each well and placed in a 37 °C constant temperature incubator for further incubation. The organoids were collected before and after 24 and 72 hours of infection with *Mtb*, washed three times with DPBS, and stored at -80 °C or 4% paraformaldehyde (Meilunbio, MA0192) for subsequent analysis.

### Acid-fast staining

LOs were dehydrated with ethanol gradient from low to high concentrations, embedded in paraffin, and cut into 4 μm thick sections. The sections were dried at 65 °C, dewaxed with ethanol from high to low concentrations, and then stained with Ziehl-Neelsen staining solution (Solarbio, G1274) at room temperature for 4 hours. Decolorization was performed with anhydrous ethanol containing 1% hydrochloric acid, and excess stain was rinsed with running water, and the cell nuclei were counterstained with hematoxylin (Solarbio, G1120). Finally, the sections were sealed with neutral resin glue (Solarbio, G8590). Images were acquired using a Vectra microscope.

### Immunofluorescence analysis

For immunofluorescence staining, sections underwent antigen retrieval with citrate buffer (Biosharp, BL04A) and were subsequently blocked with 10% goat serum (Gibco, 16210072) for 1 hour. Sections were then incubated with primary antibodies (listed in **Supplementary Table 1**) overnight at 4 °C. The following day, after washing, the sections were incubated with Alexa Fluor-conjugated secondary antibodies (Thermo Scientific) for 1 hour in the dark, and counterstained with DAPI (Cell Signaling Technology, 4083S) for 15 minutes. Finally, the sections were mounted with an anti-fade mounting medium (Dako, S3023) and imaged using an inverted or confocal laser scanning microscope (Nikon, A1R).

### CFU enumeration

LOs were collected at days 0, 1, and 3 post-*Mtb* infections, washed three times with PBS, and homogenized in PBS containing 0.05% Tween 20. The homogenates were serially diluted and plated in duplicate onto Middlebrook 7H10 agar plates. Following incubation at 37 °C for three weeks, *Mtb* colonies were enumerated.

### ATP measurement

Use a CellTiter-Glo^®^ 3D Assay kit (G9681, Promega) to detect the ATP level of LOs. Prepared a serials suspension of a 10 nM-10 μM ATP standard sample. Resuspend the above infected LOs in 100 μL medium, then plate them into a white opaque 96-well plate. Add equal volume of CellTiter-Glo^®^ 3D Reagent into standard sample or LOs, and repeatedly pipette to disperse the organoids until the LOs form into cell suspension. After Incubating at room temperature for 30 minutes, then detect the luminescence value of organoid using a GloMax^®^ Discover microplate reader (GM3000, Promega). Finally, the ATP content was calculated substituting the standard curve formula.

### Reverse transcription quantitative PCR

To quantify mRNA expression changes in LOs, RT-qPCR was conducted with TB Green Premix Ex Taq™ (RR420A, Takara) as per the manufacturer’s protocol. β-Actin served as the endogenous reference for normalization, and the 2^−ΔΔCT^ method was used to determine differential gene expression. All primer sequences are provided in **Supplementary Table 2**.

### Masson’s trichrome staining

Following deparaffinization, sections were treated with potassium dichromate overnight and subsequently rinsed under tap water. The staining procedure was then performed as follows: incubation in Weigert’s iron hematoxylin solution for 3 minutes, rinsing in running water, differentiation in 1% acid-alcohol for 10–15 seconds, and treatment with Masson’s bluing solution followed by a deionized water rinse. Subsequently, sections were stained with Ponceau-acid fuchsin for 5 minutes, differentiated in phosphomolybdic acid, and counterstained with aniline blue. After a final deionized water rinse, sections were placed in an acetic acid working solution for 1 minute. Finally, they were rapidly dehydrated through a graded ethanol and xylene series and mounted with resinous medium. Image analysis was conducted using ImageJ (Fiji, v1.8.0; NIH).

### TUNEL assay

Following dewaxing, apoptosis in organoid sections was assessed using a TUNEL apoptosis detection kit (Beyotime, C108) according to the manufacturer’s instructions. TUNEL-positive nuclei were visualized by fluorescence microscopy, and the apoptosis rate was quantified using ImageJ software (Fiji, version 1.8.0, National Institute of Health).

### Western blot

RIPA lysis buffer (MedChemExpress, HK-K1001) supplemented with a protease and phosphatase inhibitor cocktail (MedChemExpress, HY-K0021) was added to the LOs stored at -80°C by repeated pipetting, and then the organoid samples were lysed on ice. After sufficient lysis, the samples were centrifuged at 12,000×g for 10 minutes at 4°C. The supernatant was collected, 5×SDS Loading buffer (Solarbio, P1040) was added and boiled to denature the protein, and electrophoresed on a 10% SDS-PAGE gel (Vazyme, E303-01) at 120V, and then the protein was transferred to a PVDF membrane and blocked with TBST (Servicebio, G0004-1L) containing 5% skim milk (Beyotime, P0216-300g) for 1 hour. After incubation with primary antibodies at 4°C overnight, proteins were incubated with corresponding secondary antibodies for 1 hour at room temperature. Western blots were visualized using the ChemiDOC XRSP system (BioRad). The primary antibodies are listed in **Supplementary Table 1**.

### Enzyme-Linked Immunosorbent Assay

The supernatant of LOs infected with *Mtb* was collected and centrifuged at 2000×g for 20 minutes, and 50 µL was used for enzyme-linked reaction. According to the manufacturer’s instructions, the secretion levels of IL-8 (D8000C, R&D), IL-1β (DY401, R&D), and MCP-1 (DCP00, R&D) were detected.

### Transcriptome analysis

RNA was extracted from LOs using Trizol extraction kit (Gibco, 15596026CN). 1 μg of total RNA was used for library preparation. Oligo (dT) beads were used for isolation of poly (A) mRNA. mRNA fragmentation was performed under divalent cation and high temperature conditions. Primers were prepared using random primers. First-strand cDNA and second-strand cDNA were synthesized. The purified double-stranded cDNA was then treated to repair both ends and add a da tail in one reaction, followed by T-A ligation to add adapters on both ends. The adapter-ligated DNA was then size-selected using DNA cleaning beads. PCR amplification was performed using P5 and P7 primers, and PCR products were verified. Libraries with different indexes were then multiplexed and loaded onto Illumina HiSeq/Illumina Novaseq/MGI2000 instruments for sequencing using 2×150 paired-end (PE) configuration according to the manufacturer’s instructions. Raw sequencing reads were processed and aligned to the reference genome. Differential expression analysis was performed using DESeq2. Genes with an adjusted p-value (false discovery rate, FDR) < 0.05 and an absolute log_2_ fold change (|log_2_FC|) > 1 were considered statistically significant. These thresholds are consistently applied and reported in the Results section. Enrichment analysis of Gene Ontology (GO) terms and Kyoto Encyclopedia of Genes and Genomes (KEGG) pathways was performed on these differentially expressed gene sets. The transcriptome sequencing data described above has been deposited in the National Genomics Data Center (NGDC), and the dataset accession number is HRA011958.

### Statistical analysis

All quantitative data are presented as mean ± SEM (standard error of the mean). The sample size (n) for each experiment is specified in the corresponding figure legend and represents the number of biologically independent organoids or replicates. Image-based quantifications were performed in a blinded manner where applicable. For comparisons between two groups, statistical significance was assessed using an unpaired two-tailed Student’s t-test. For comparisons across more than two groups (e.g., multi-timepoint infection experiments), one-way analysis of variance (ANOVA) followed by Tukey’s *post hoc* test was used. A p-value < 0.05 was considered statistically significant. All statistical analyses were performed using GraphPad Prism software (version 9.0.0).

## Results

### Generation of human lung organoids

**Figure 1:**
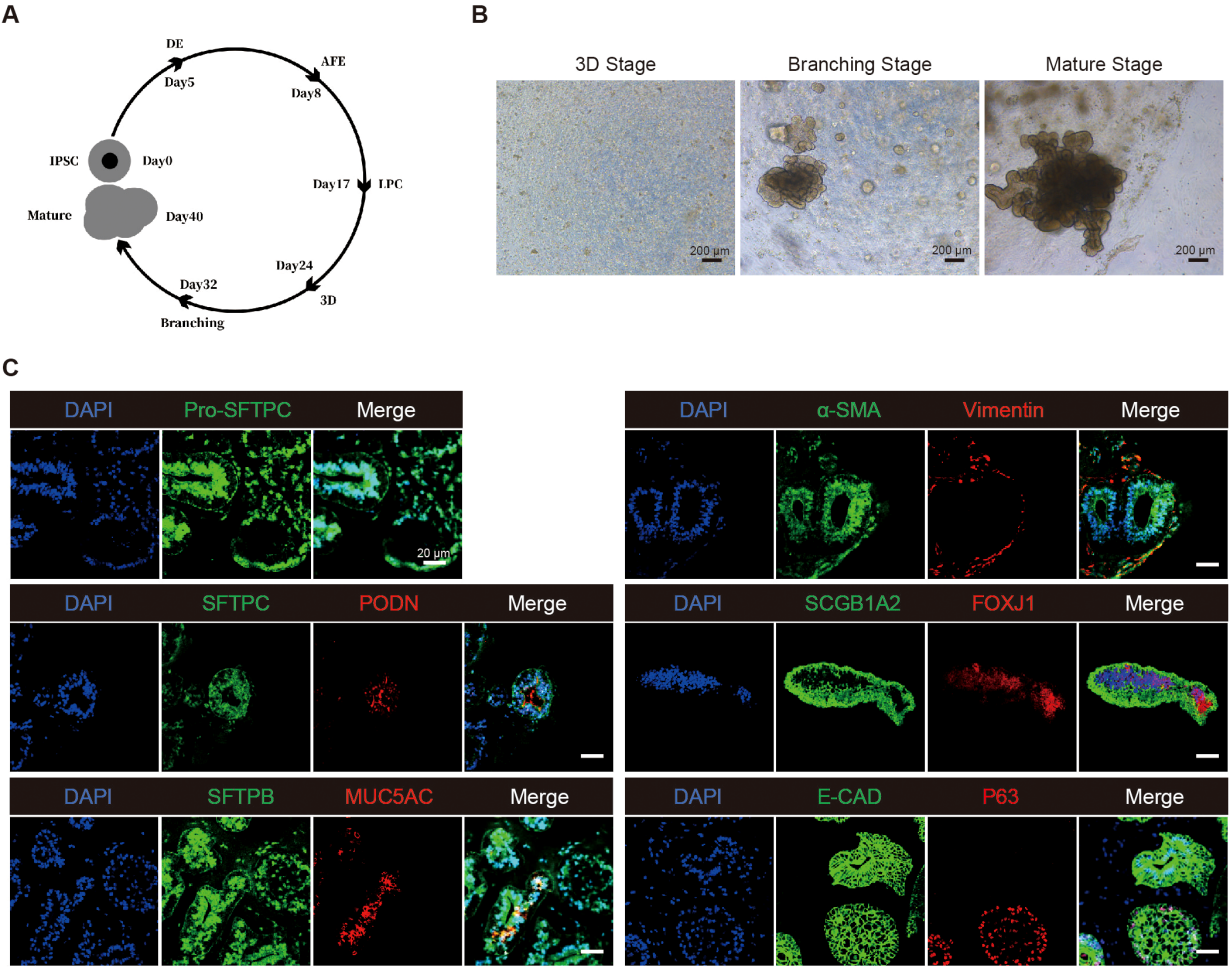
Generation of human iPSCs-derived lung organoids (LOs). **(A)** Schematic representation of the protocol for differentiating iPSCs into mature LOs. **(B)** Representative images of lung progenitor cells differentiating toward mature lung organoid. Scale bar, 200 μm. **(C)** The immunofluorescence identification image of LOs. DAPI, cell nuclei; SFTPB, Pro-SFTPC, SFTPC: type II alveolar; PODN, type I alveolar cells; MUC5AC, proximal airway epithelial cells; α-SMA:fibroblasts; Vimentin, marker for stromal (or interstitial) cells; SCGB3A2, secretory cells; FOXJ1, ciliated cells; E-CAD, epithelial cells; P63, basal cellsl; Scale bar, 20 μm.

To establish an *in vitro* model of pulmonary TB, LOs derived from human iPSCs were prepared based on the protocol previously reported (Leibel et al., 2019; Leibel et al., 2021). Human iPSCs were successfully differentiated into LOs through sequential stages of definitive endoderm (DE), anterior foregut endoderm (AFE), and lung progenitor cell (LPC). The cells at the LPC stage were then embedded in Matrigel and cultured in 3D format in 6-well plates (**Figure 1A**). After 14 days of cultivation, primitive LOs aggregates, predominantly comprising thin-walled hollow spherical structures, were observed in the Matrigel under microscopic examination (**Figure 1B**).

When treated with Branching Medium (containing FGF7 and FGF10) and Mature Medium, the organoid spheres underwent rapid expansion. Following this initial expansion, the combined action of retinoic acid (RA), dexamethasone (DEX), cyclic AMP (cAMP), and 3-isobutyl-1-methylxanthine (IBMX) promoted functional maturation, leading to the development of large, mature organoids with vacuolar, alveolar-like structures by day 40 (**Figure 1B**). Immunofluorescence analysis indicated the presence of multiple proximal airway epithelial cells, such as club cells, ciliated cells, and goblet cells, as well as distal airway epithelial cells, including type I and type II alveolar epithelial cells (**Figure 1C**). Given that organoids exhibit a reversed polarity configuration, with the apical surface of the airway epithelium facing inward and the basolateral surface facing outward, this model may more effectively simulate the susceptibility of basally exposed epithelial cells to *Mtb* infection under conditions of airway epithelial injury—a clinically relevant route of infection (Jakiela et al., 2008). Additionally, the LOs also express the basal cells and myofibroblasts. The co-existence of proximal and distal lineages, along with mesenchymal cells within the same organoid, reflects the proximal-distal patterning observed in the developing human lung, thus confirming their faithful representation of the cellular composition and organizational principles of the human lung.

### *In vitro* modeling of pulmonary TB infection

**Figure 2:**
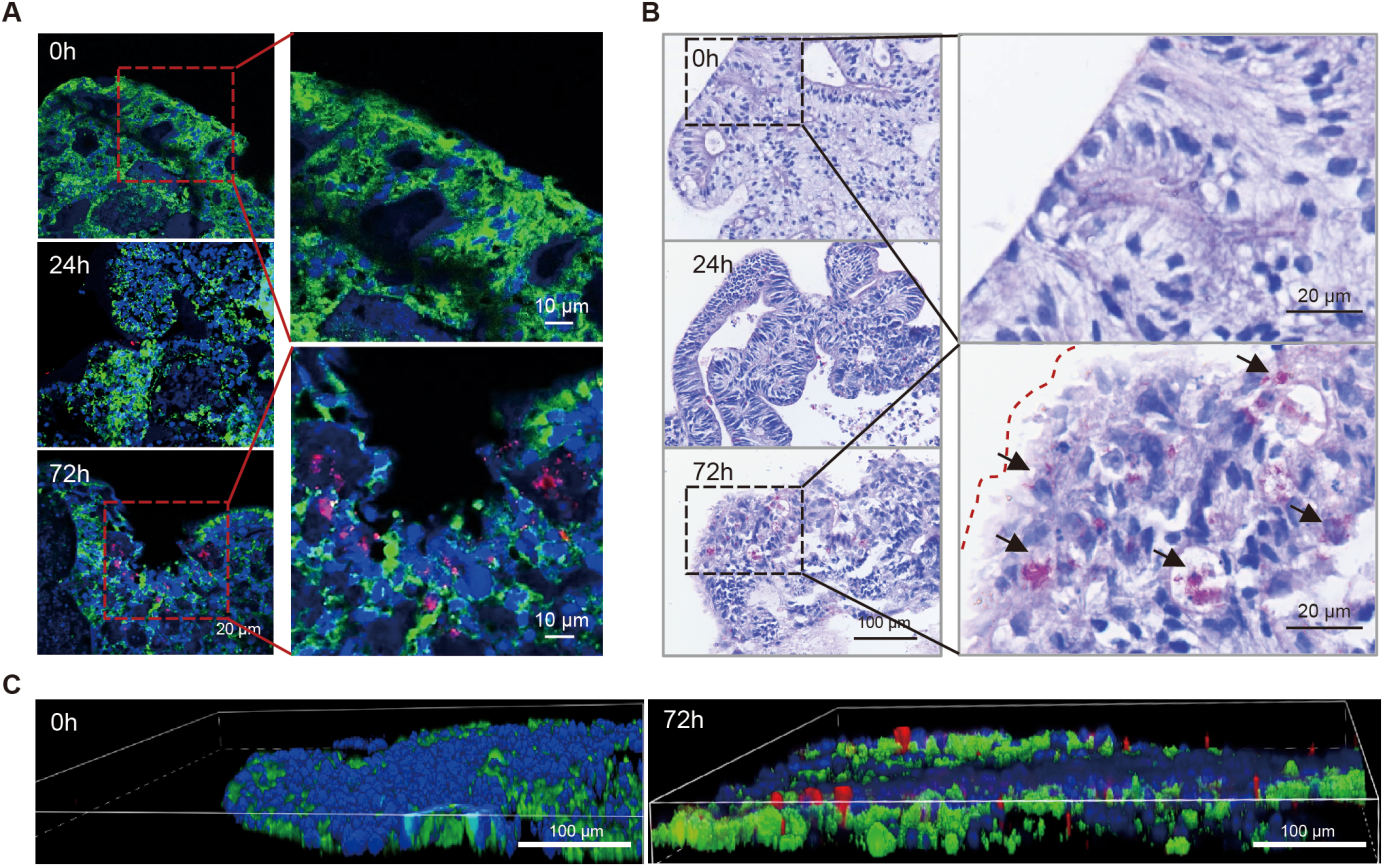
Generation of pulmonary TB *in vitro* model. Immunofluorescence **(A)** and acid-fast staining **(B)** images of *Mtb*-infected LOs at indicated days post-infection. Scale bar=10 µm, 20 µm or 100 µm, as indicated in the image. **(C)** Representative 3D immunofluorescence reconstruction images of *Mtb*-infected LOs at 0 and 3 days post-infection. *Mtb* is shown in red, actin filaments are in green, and nuclei is in blue, Scale bar, 100 µm.

To assess their susceptibility against *Mtb*, LOs were released from Matrigel and transferred to ultra-low attachment plates for direct inoculation with *Mtb.* At the early stages of infection, immunofluorescence and acid-fast bacilli (AFB) staining revealed organoids with intact peripheral structure and no detectable *Mtb* (**Figures 2A, B**). After 24 hours, AFB-stained *Mtb* with red fluorescence accumulated at the organoid periphery. By 72 hours, the bacteria had successfully penetrated the organoids, as evidenced by the presence of numerous red-stained bacilli in their interior (**Figure 2C**).

**Figure 3:**
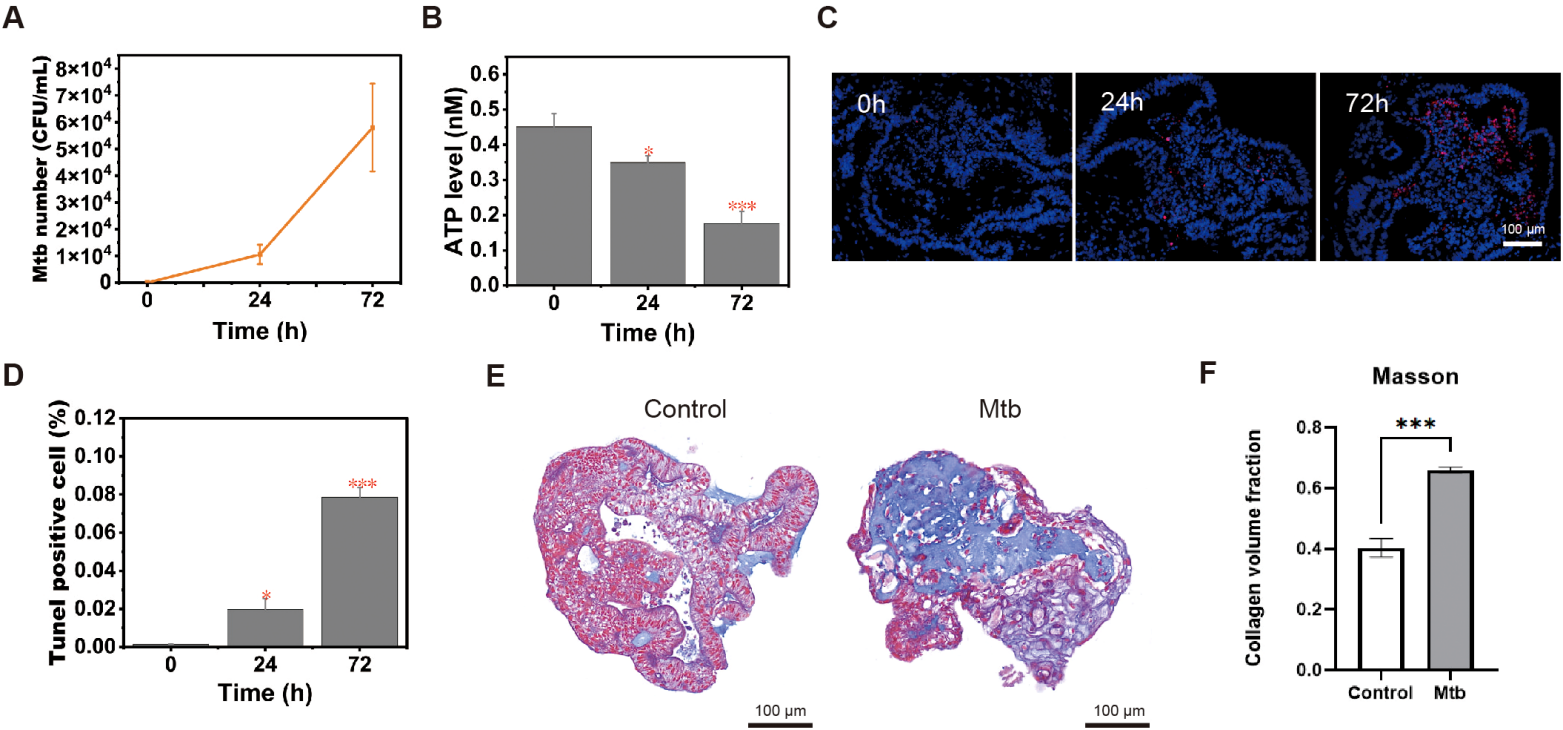
*Mtb* infection induces damage to LOs. **(A)** Quantitative analysis of bacterial load at a specified time points post-infection (0, 24 and 48 hours). Data are presented as mean ± SEM from n=3 independent biological replicates. Statistical significance was assessed by an unpaired two-tailed Student’s t-test. **(B)** Intracellular ATP levels were measured in *Mtb*-infected LOs models at 3 days post-infection. Data are mean ± SEM (n = 8 organoids per group from 3 independent differentiations). One-way ANOVA with Tukey’s *post-hoc* test was performed. *p < 0.05, ***p<0.001. **(C, D)** Apoptotic cells in *Mtb*-infected LOs **(C)** and quantitative analysis **(D)**. Scale bar=100 μm. (n = 5 fields from 3 independent organoid samples) **(E, F)** Representative images of Masson’s trichrome staining in LOs with or without *Mtb* infection **(E)** and quantitative analysis **(F)** of collagen content in the LOs pre- and post-*Mtb* infection. Scale bar=100µm, n = 5 fields from 3 independent organoid samples.

The disruption of organoid edges and the presence of apoptotic bodies upon hematoxylin staining indicated *Mtb*-induced barrier destruction (**Figure 2B**). Quantification of the internal bacterial load upon LOs lysis showed a 4-5-fold increase in *Mtb* numbers from day 1 to day 3 (**Figure 3A**). In addition, assessment of organoid viability using an ATP detection kit revealed a more than 50% reduction in viability by day 3 due to the increased bacterial content (**Figure 3B**). TUNEL staining demonstrated an increase in apoptotic cells on day 3 (**Figures 3C, D**), distributed not only near direct bacterial contact but also present in internal regions. Furthermore, Masson’s trichrome staining showed a general rise in total collagen levels in *Mtb*-infected LOs in contrast to uninfected controls, suggesting that *Mtb* infection initiates early profibrotic extracellular matrix remodeling, leading to collagen deposition (**Figures 3E, F**). Collectively, these results demonstrate that the infected LOs recapitulate key pathological features of TB, providing experimental evidence for their utility in modeling the disease.

### Transcriptome analysis gene expression and molecular pathway alterations in LOs during the early stages of *Mtb* infection

**Figure 4:**
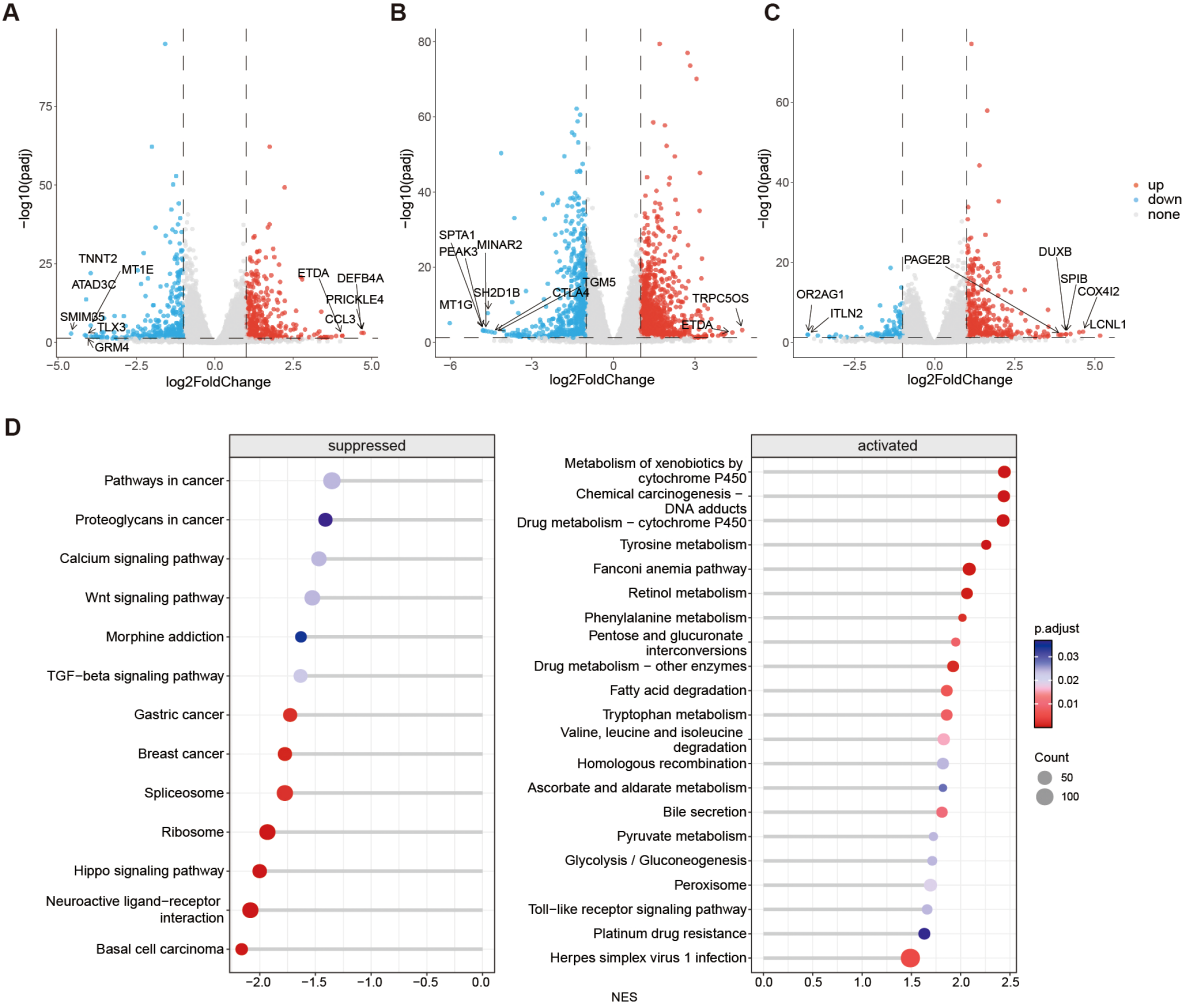
Transcriptomic profiling of LOs at different time points after *Mtb* infection. **(A-C)** Volcano plots of differentially expressed genes (DEGs) identified from RNA-seq analysis. Comparisons were made between **(A)** LOs at 1 day post-infection *vs.* uninfected controls, **(B)** 3 days post-infection *vs.* uninfected controls, and **(C)** 3 days post-infection *vs.* 1 day post-infection. DEGs were defined by an adjusted p-value (FDR) < 0.05 and |log_2_ fold change| > 1 (determined by DESeq2 or appropriate statistical test) are considered significant. Significantly upregulated genes are highlighted in red, and downregulated genes are highlighted in blue. Data are representative of n=3 independent biological replicates per condition. **(D)** Kyoto Encyclopedia of Genes and Genomes (KEGG) pathway enrichment analysis for the DEGs identified in the 1 day post infection *vs.* uninfected control [from panel **(A)**]. The top significantly enriched pathways are shown. Enrichment significance was calculated using a hypergeometric test/Fisher’s exact test, with a corrected p-value (Benjamini-Hochberg) < 0.05 considered significant. The bubble plot depicts the enrichment score (-log10(p-value)) and the number of genes associated with each pathway.

Transcriptome sequencing was conducted on LOs to investigate early molecular events during *Mtb* infection. Differential gene expression analysis was performed comparing uninfected organoids to those infected for 1 and 3 days. Volcano plots illustrate the distinct gene expression profiles. Genes with a p-value < 0.05 and fold change > 1.0 were considered differentially expressed (**Figures 4A–C**). Upregulated genes, including *CCL3*, *DEFB4A*, *ETDA*, and *PRICKLE4*, were identified post-infection. *CCL3*, a chemokine involved in leukocyte chemotaxis, plays a crucial role in initiating immune response. *DEFB4A* is a member of the β-defensin family. *ETDA* and *PRICKLE4* associated with organ morphological development. Nevertheless, the specific roles of these highly expressed genes following *Mtb* infection remain unclear.

Downregulated genes identified in *Mtb*-infected LOs were *TNNT2*, *ATAD3C*, *MT1E*, *TLX3*, *SMIM35*, and *GRM4*. Variances in gene expression were observed between Day 3 versus Day 1 post-infection. The volcano plot revealed upregulation of genes such as *PAGE2B*, *DUXB*, *SPIB*, *COX4I2*, and *LCNL1*, associated with the P-antigen family, gene transcription regulation, and metabolism. Conversely, downregulated genes exhibited a modest decrease, with significant reductions in expression levels of *OR2AG1* and *ITLN2*. Additionally, comparison between the 3-day infection group and the uninfected group also revealed the upregulation of *TRPC5OS* and *ETDA* genes, along with the downregulation of multiple genes such as *SPTA1* and *PEAK3*. While their precise biological functions under *Mtb* infection require further validation.

Kyoto Encyclopedia of Genes and Genomes (KEGG) enrichment analysis revealed that pathways associated with the early stage of *Mtb* infection at day 1 primarily Drug metabolism-cytochrome P450, Tyrosine metabolism, Retinol metabolism, Phenylalanine metabolism, Pentose and glucuronate interconversions, Fatty acid degradation, Pyruvate metabolism, Glycolysis/Gluconeogenesis, Chemical carcinogenesis-DNA, Homologous recombination, Toll−like receptor signaling pathway, and Herpes simplex virus 1 infection (**Figures 4D**). These pathways suggests that LOs may experience metabolic stress and activate some immune responses during the early stages of *Mtb* infection.

**Figure 5:**
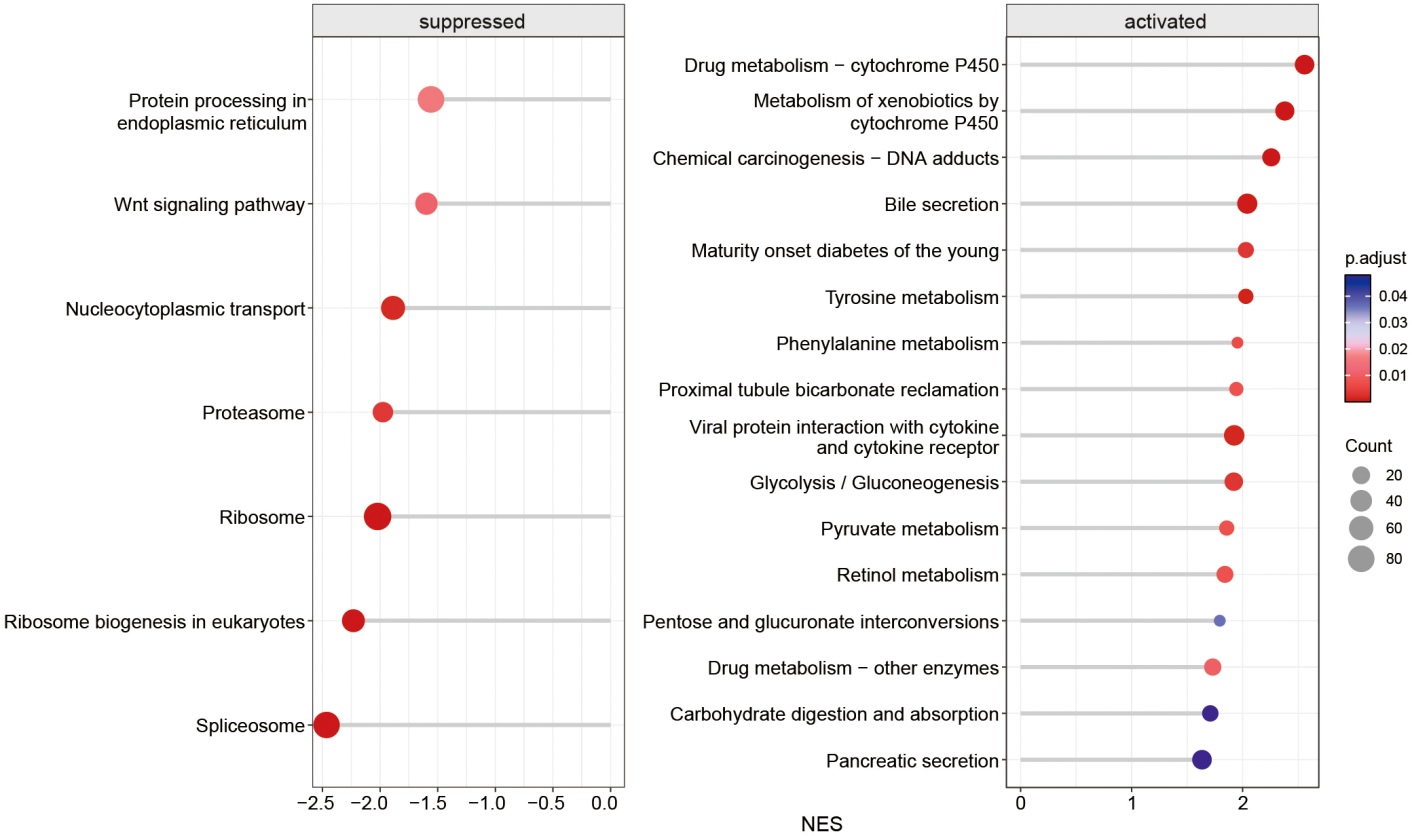
KEGG pathways enriched by differentially expressed genes in LOs infected with *Mtb* after 3 days.

After 3 days of *Mtb* infection, the enriched upregulated pathways in the KEGG database primarily involve substance metabolism, biosynthesis, DNA repair, inflammatory responses, and energy metabolism (**Figure 5**). Conversely, the downregulated KEGG terms predominantly affect essential processes such as intracellular protein processing, signal transduction, nucleocytoplasmic transport, protein degradation, protein synthesis, and mRNA splicing (**Figure 5**). The downregulation of these processes suggests impaired functionality, potentially impacting the normal growth, development, and metabolism of LOs.

### Temporal gene expression dynamics and signaling pathway alterations during early infection stages

**Figure 6:**
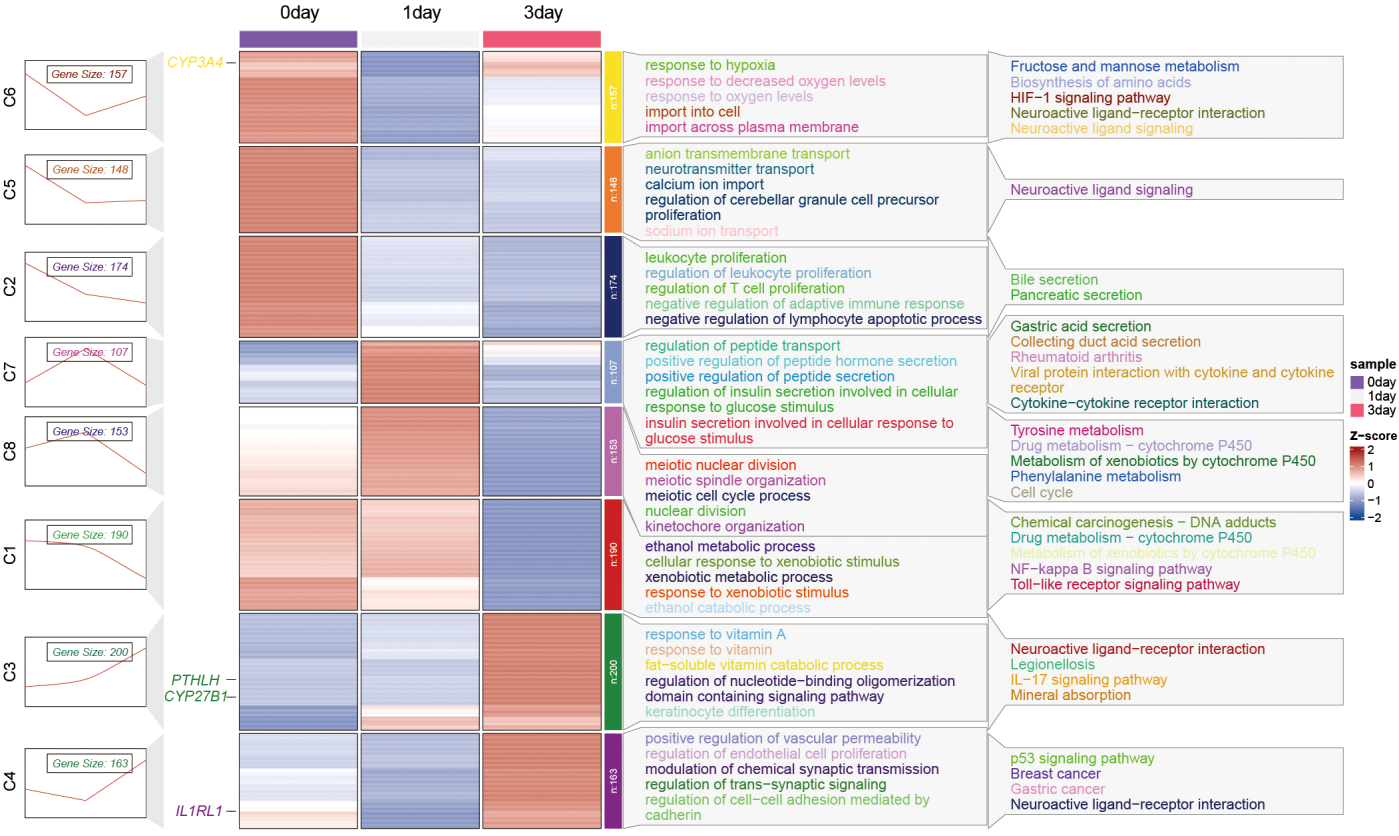
Unsupervised clustering of DEGs (FDR < 0.05 and |log_2_FC| > 1) from LOs at 1 and 3 days post-*Mtb* infection compared to uninfected controls revealed eight distinct temporal expression patterns (C1-C8) using the Mfuzz algorithm. n = 3 biologically independent replicates per condition. Subsequent functional enrichment analysis identified the predominant biological themes for each cluster: Gene Ontology (GO) terms are listed on the left, and Kyoto Encyclopedia of Genes and Genomes (KEGG) pathways on the right.

Temporal trend analysis of gene expression differences at 1- and 3-days post-infection was conducted using the Mfuzz package (time trend analysis). Differentially expressed genes were categorized into eight subsets (C1-C8) based on KEGG (right pathway) and GO data (left pathway) classifications (**Figure 6**). Subsets C3, C4, and C6 consistently exhibited upregulation or downregulation following infection, suggesting their continuous involvement in relevant biological processes during early infection stages. Subset C3 exhibited activation of pathways including the NF-κB signaling pathway, Metabolism of xenobiotics by cytochrome P450, etc. Subset C4 displayed upregulation of pathways related to energy metabolism and absorption such as Protein digestion and absorption following infection. In contrast, subset C6 showed downregulation of pathways like Neuroactive ligand-receptor interaction, IL-17 signaling pathway, etc. Collectively, the modulation of these pathways-particularly NF-κB, IL-17, and neuroactive ligand interactions-suggests a coordinated reprogramming of innate immunity and cytokine signaling in the LOs during the initial stages of *Mtb* infection.

**Figure 7:**
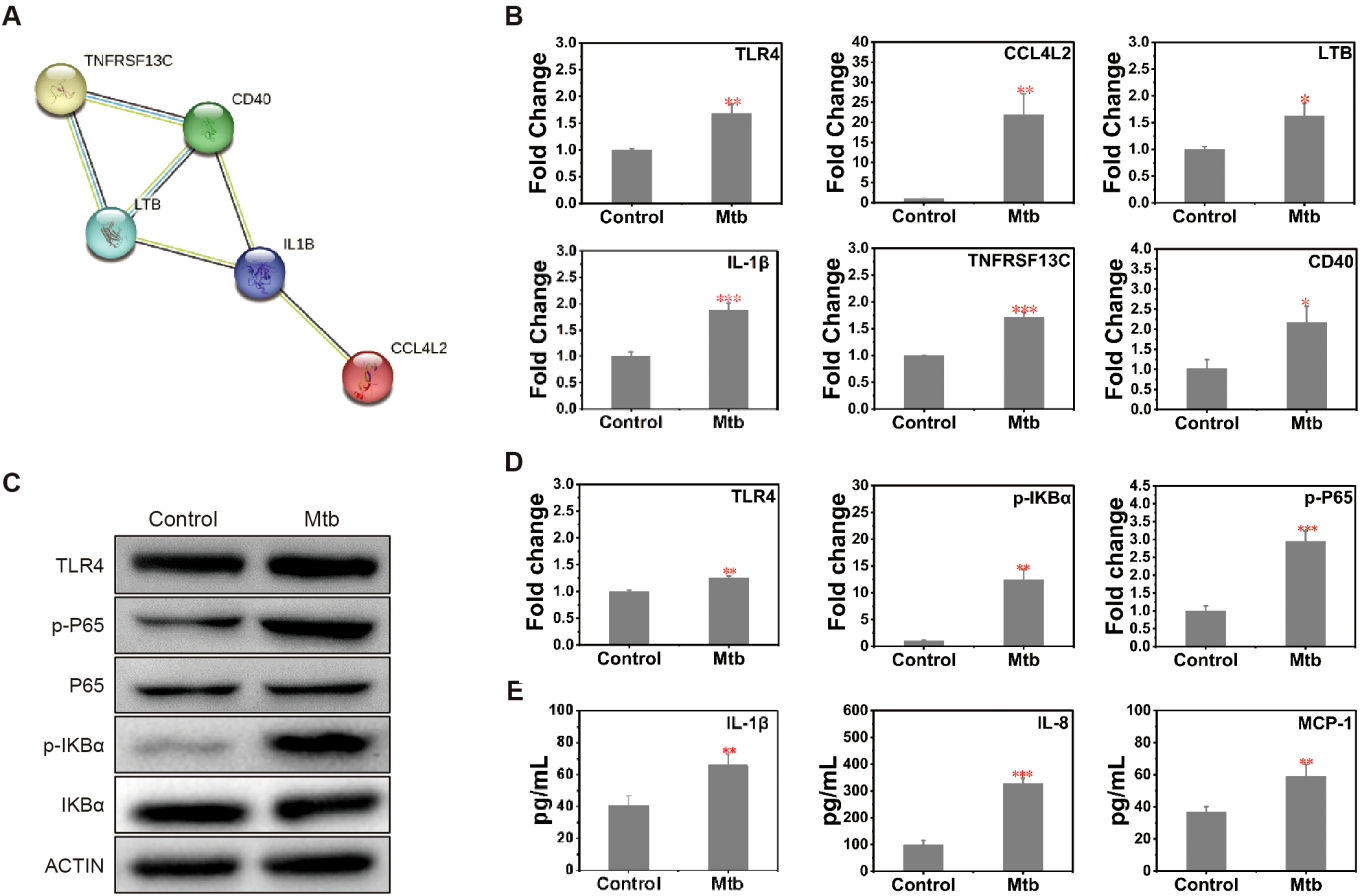
Activation of the TLR4-NF-κB signaling pathway and induction inflammatory cytokine response in *Mtb-*infected LOs. **(A)** The protein-protein interaction (PPI) network of genes upregulated in the NF-κB signaling pathway was identified from transcriptomic data. This network was constructed using the STRING database and visualized in Cytoscape. **(B)** Quantitative PCR (qPCR) analysis of the mRNA expression levels of key genes in the NF-κB signaling pathway in LOs both pre- and post-*Mtb* infection. Gene expression was normalized to *ACTIN* and is presented as mean ± SEM from n=3 independent biological replicates. Statistical significance was determined using an unpaired two-tailed Student’s t-test (*p < 0.05, **p < 0.01, ***p<0.001). **(C, D)** Western blot analysis results show the expression levels **(C)** and quantitative analysis **(D)** of key genes in the TLR4-NF-κB signaling pathway in LOs pre- and post-*Mtb* infection. **(E)** ELISA analysis of inflammatory cytokines IL-1β, IL-8, MCP-1 secreted in the supernatant of LOs both pre- and post-*Mtb* infection, n=3.

TLR4 activates NF-κB signaling and induces inflammation in innate immunity upon sensing danger signals from *Mtb*, initiating the innate immune response. A Protein-Protein Interaction network analysis was performed for the upregulated genes within the NF-κB pathway (**Figure 7A**). Furthermore, the activation process of the NF-κB signaling pathway was validated by qPCR. Following infection, immune response-related genes such as *TLR4, CCL4L2*, *TNFRSF13C*, *CD40*, *IL-1β*, and *LTB* showed upregulated expression (**Figure 7B**). These results are consistent with the upregulation of TLR4 and the accompanying changes in the NF-κB signaling pathway, suggesting that this pathway may be activated in the early stages of *Mtb* infection.

The KEGG pathway analysis demonstrates a coordinated transcriptional response to *Mtb* infection. Our KEGG enrichment analysis collectively illustrates a change in the transcriptional profile of *Mtb*-infected LOs, characterized by an increase in metabolic and innate immune pathways and a decrease in pathways related to development and signal transduction. These coordinated changes suggest a shift in host cell priorities toward defense and metabolism, the functional implications of which represent a compelling direction for future research.

### *Mtb* infection activates NF-κB pathway and induces inflammatory cytokine response in LOs

The protein expression and phosphorylation status of key genes in the pathway were further investigated by examining NF-κB activation in infected and uninfected organoids at 3 days post-infection. Upregulation of TLR4 protein post-infection was confirmed. While no significant differences were observed in the total protein expression levels of NF-κB, P65 and the signaling activation protein IKB-α, their phosphorylated forms exhibited upregulation (**Figures 7C, D**). As a key downstream effector of NF-κB signaling, the production of pro-inflammatory cytokines was assessed. ELISA was utilized to detect inflammatory cytokines in the supernatants of *Mtb*-infected LOs. Following infection, elevated levels of IL-1β, a pyrogen and central mediator of inflammation; IL-8, a potent neutrophil chemoattractant; and MCP-1, a key recruiter of monocytes, were detected (**Figure 7E**). These findings suggest that *Mtb* infection is associated with TLR4 upregulation and activation of the NF-κB pathway, subsequently influencing downstream inflammatory responses.

## Discussion

Several studies have shown that *Mtb* disrupts the barrier function of the alveolar epithelium, potentially aiding its penetration. This disruption likely involves the breakdown of tight junction structures induced by *Mtb* (Zang et al., 2024). Monolayer epithelial cell models are limited in simulating this transmission process due to their lack of complex cellular architecture and intercellular junctions (Leestemaker-Palmer and Bermudez, 2023). Animal models provide limited insight due to the species-specific tropism of *Mtb* for humans, such as the fundamental differences in immune responses and lung lobe structure in mice (Rydell-Törmänen and Johnson, 2019; Kim et al., 2024). Animal models are valuable for modeling different aspects of human TB. Guinea pigs, for example, are highly susceptible to airborne *Mtb* and are used to study transmission, while rabbits exhibit human-like cavitary lesions (Singh and Gupta, 2018; Yang et al., 2021). However, as animals are not natural hosts for *Mtb*, these models only partially reproduce clinical and immunological features of human TB and often differ in granuloma formation and disease susceptibility (Orme, 2003; Flynn et al., 2015; Fonseca et al., 2017; Singh and Gupta, 2018). Therefore, there is an urgent need to establish a highly human-relevant *in vitro* model to bridge the gaps in these existing model systems and more accurately investigate the pathogenesis of human TB. Human LOs, which replicate the cellular composition and function of the lungs, have emerged as outstanding models for investigating pathophysiology and drug screening (Leon-Icaza et al., 2025; Li et al., 2025; Rothan et al., 2025; Ryu et al., 2025). These organoids, serving as reliable and physiologically relevant human models, offer significant insights into the exploration of various stages of TB activity (Tan et al., 2019; Kim et al., 2024; Zhang et al., 2025). Unlike studies focused on direct infection of the apical airway epithelium (Kim et al., 2024), our findings demonstrate that *Mtb* can adhere to and survive within the basally exposed surfaces of LOs. This reveals a distinct, non-canonical route of infection and suggests that epithelial structures, in addition to professional phagocytes, could facilitate bacterial transit across the mucosal barrier. We acknowledge that this configuration differs from the initial aerosol exposure; however, it effectively models the critical post-barrier disruption stage, which is a pathophysiologically relevant scenario involving basolateral access via paracellular routes or protease-mediated junction degradation (Zang et al., 2024).

Beyond modeling bacterial invasion, our LOs system recapitulates the key pathological features characterized by epithelial cell death and collagen deposition, which were insufficiently addressed in the previous report (Iakobachvili et al., 2022), but mirrors the characteristics of pro-fibrosis pathology commonly observed in patients (González-Avila et al., 2009). The upregulation of collagen genes is implicated in the progression of pulmonary fibrosis, leading to impaired lung function. Consistent and quantifiable increases in collagen content were observed across replicates through the detection of fibrosis-related gene expression. However, our data primarily capture this early phenotypic change post-infection. Given the multicellular complexity of LOs, we cannot yet distinguish whether collagen is derived from resident mesenchymal fibroblasts or from epithelial cells undergoing Epithelial-Mesenchymal Transition (EMT), although both likely contribute. Identifying the specific cellular sources of collagen is a critical direction for future research utilizing this model. Future studies utilizing extended infection time courses will be essential to fully elucidate the dynamics of fibrotic progression in this human-relevant model. This will provide a more comprehensive understanding of the long-term tissue remodeling events associated with chronic *Mtb* infection. Besides focusing solely on the intrinsic functions of LOs, future optimization efforts could also draw upon the advantages of lung-on-chip systems, which can effectively reconstitute immune-epithelial interactions under physiologically relevant mechanical cues (Luk et al., 2026). Hence, these findings suggest that LOs may serve as a novel platform for developing host-directed therapies targeting fibrosis (Wallis and Hafner, 2015). Besides, in response to bacterial invasion, LOs mount a coordinated defense by activating various immune signaling pathways, including the secretion of antimicrobial peptides (De Waal et al., 2022). Our study identified the upregulation of DEFB4A, a defensin type with potent bactericidal properties, suggesting its potential as a novel therapeutic approach.

To recognize *Mtb* and its products for initiating signal transduction and recruiting other immune cells, airway and alveolar epithelial cells exhibit responsiveness to pathogen-associated molecular patterns (PAMPs) and danger-associated molecular patterns (DAMPs) *via* Pattern Recognition Receptors (PRRs) (Whitsett and Alenghat, 2015; Constant et al., 2021). Toll-like receptors (TLRs), a subset of PRRs, are crucial in activating the NF-κB signaling pathway upon *Mtb* infection (Kawai and Akira, 2007; Zhang et al., 2015; Hadifar et al., 2019). TLR2 and TLR4 are among the TLRs expressed by airway epithelial cells and type II alveolar epithelial cells (Wu et al., 2011; Godbole et al., 2022). Activation of the TLR4-NF-κB signaling pathway leads to the production of downstream cytokines and chemokines (Vivekanandan et al., 2023). RNA-sequencing analysis revealed elevated levels of inflammatory cytokines in LOs during *Mtb* infection. Additionally, DEFB4A upregulation, a downstream gene of the NF-κB pathway (De Waal et al., 2022), was observed, along with increased expression of chemokines such as IL-1β, MCP-1, and IL-8. This data demonstrates a potential activation of TLR4-NF-κB pathway, which may contribute to the release of subsequent cytokine and chemokine. Further investigations, including pathway-specific inhibition experiments, are necessary to clarify the pivotal role of this signaling axis in LOs’ response to *Mtb* infection.

Several technical limitations exist in this study. For instance, there is a short infection window (≤72 hours), lack of confirmation of bacterial viability post-infection (CFU not extended beyond day 3), absence of mechanistic inhibitors for NF-κB or TLR4 to validate causality, potential polarity mismatch (apical *versus* basolateral exposure) that could alter PRR engagement and physiological relevance, reliance on a single or limited number of iPSC lines limiting generalizability, and unresolved differentiation between extracellular and intracellular *Mtb*. Future studies aim to achieve apical-out polarity via microinjection or suspension culture strategies to establish an apical infection model, allowing for more physiologically relevant study of host-pathogen interactions.

Above all, an *Mtb* infection model was developed using human iPSCs-derived LOs, which reveals early host-pathogen interactions. Our findings indicate that LOs can internalize *Mtb* and elicit an innate immune response characterized by the upregulation of TLR4 and activation of the NF-κB signaling pathway, resulting in the secretion of pro-inflammatory cytokines. Despite demonstrating substantial physiological relevance, LOs model’s limitations still require further refinement. For instance, the model lacks immune components and vascular network, impede the investigation of immune cell trafficking and bacterial systemic dissemination. As a result, the cytokines and fibrotic response observed post *Mtb* infection solely reflect the intrinsic pathways of epithelial cells. While the model effectively delineates the pulmonary epithelium’s role in initial infection stages, it fails to recapitulate the complete spectrum of granuloma formation or the chronic phase of TB. To overcome these limitations, researchers have employed co-culture methods with various cell types including endothelial cells, mesenchymal cells, and immune cells, and incorporated bioengineering platforms to more closely mimic the *in vivo* microenvironment (Kang et al., 2025; Li et al., 2025; Zhang et al., 2025). These advancements will facilitated applications of LOs to test innovative therapeutic strategies targeting host as well bacteria, thereby providing insights into disease mechanisms and potential treatments. 

## Data Availability

The datasets presented in this study can be found in online repositories. The names of the repository/repositories and accession number(s) can be found below: https://ngdc.cncb.ac.cn/gsa-human/s/D7x125v4, HRA011958.
