# Supplementary material for: Human lung organoids model for assessing host response to Mycobacterium tuberculosis infection
Source: Front Cell Infect Microbiol. 2026 Feb 10;16:1725344. doi: 10.3389/fcimb.2026.1725344 (PMC12929407; doi:10.3389/fcimb.2026.1725344)
Supplement: Supplementary file 1 [file Table1.docx]

Supplementary Table 1. Antibody used in this study

| Primary antibodies | Cat# | Source | Dilution ratio |
| --- | --- | --- | --- |
| DAPI | 4083S | CST | IF: 1:2000 |
| SFTPB | MA1-204 | Thermo | IF: 1:100 |
| Pro-SFTPC | ab196677 | Abcam | IF: 1:50 |
| SFTPC | sc-518209 | Santa Cruz | IF: 1:250 |
| PODN | ab10288 | Abcam | IF: 1:500 |
| MUC5AC | ab198294 | Abcam | IF: 1:250 |
| α-SMA | ab5694 | Abcam | IF: 1:100 |
| Vimentin | ab8978 | Abcam | IF: 1:500 |
| SCGB3A2 | ab181853 | Abcam | IF: 1:500 |
| FOXJ1 | 14-9965-82 | Invitrogen | IF: 1:250 |
| E-CAD | 610181 | BD | IF: 1:50 |
| P63 | ab124762 | Abcam | IF: 1:200 |
| TLR4 | ab13556 | Abcam | WB: 1:500 |
| p-P65 | ab278777 | Abcam | WB: 1:500 |
| P65 | ab32536 | Abcam | WB: 1:500 |
| p-IKBα | ab92700 | Abcam | WB: 1:500 |
| IKBα | ab32518 | Abcam | WB: 1:500 |
| Actin | 8H10D10 | CST | WB: 1:500 |

**Supplementary Table S2: Primer sequences used in this study.**

| Target gene | Direction | Primer sequence (5’to 3’) |
| --- | --- | --- |
| TLR4 | Forward | GATAGCGAGCCACGCATTCA |
|  | Reverse | TTAGGAACCACCTCCACGCA |
| CCL4L2 | Forward | GCACAGGACACAGCTAGGTT |
|  | Reverse | GCAGACTTGCTTGCCTACCA |
| LTB | Forward | GAGGACTGGTAACGGAGACG |
|  | Reverse | AGAAACGCCTGTTCCTTCGT |
| IL-1β | Forward | TTACAGTGGCAATGAGGATGAC |
|  | Reverse | GTGGTGGTCGGAGATTCGTA |
| TNFRSF13C | Forward | TGCTCCAAGACACAGCACAT |
|  | Reverse | CAAGGGACCTCATGTCCACC |
| CD40 | Forward | AGCAGATTGGTCCCCAGGAT |
|  | Reverse | TGAGGACTCACTGATAAAGACCAG |
